# The effect of type of task on EFL learners’ vocabulary learning

**DOI:** 10.3389/fpsyg.2024.1306306

**Published:** 2024-07-05

**Authors:** Zahra Eskandari, Omid Khatin-Zadeh, Danyal Farsani, Hassan Banaruee

**Affiliations:** ^1^School of Foreign Languages, University of Electronic Science and Technology of China, Chengdu, China; ^2^Department of Teacher Education, Norwegian University of Science and Technology, Trondheim, Norway; ^3^Department of Educational Psychology, University of Education Weingarten, Weingarten, Germany

**Keywords:** technique feature analysis, task-based learning, incidental vocabulary learning, vocabulary acquisition, teaching English (as a foreign language)

## Abstract

Depth of processing vocabulary has been the subject of heated discussion among vocabulary researchers. Yet, current literature lacks research comparing different tasks to investigate the acquisition of vocabulary knowledge among adult learners of English as a foreign language (EFL). To fill the gap, we designed five task-based groups based on *Technique Feature Analysis* (TFA) as a framework to predict the effectiveness of different vocabulary learning tasks with similar or different TFA rankings on L2 vocabulary knowledge gain. The participants were 130 EFL learners (mean age = 21.7, female 61.5%) randomly assigned to the vocabulary learning tasks: reading and multiple-choice items (TFA = 6), reading and choosing definitions (TFA = 6), reading and fill-in-the-blanks (TFA = 7), reading and rewording the sentences (TFA = 6) and composition writing (TFA = 8). The results of the study revealed that tasks with the same TFA scores led to similar vocabulary knowledge gains. While predictions of the TFA are partially supported, composition writing and sentence rewording tasks supersede other tasks in terms of their effectiveness in vocabulary acquisition.

## 1 Introduction

One of the most crucial components of teaching and learning a second language is vocabulary, since without sufficient vocabulary, students cannot properly convey or understand a massage (Wilkins, 1972; Coady and Huckin, 1997; Schmitt, 2008; Macedonia, 2014; Macedonia et al., 2014). On one hand, “without vocabulary nothing can be conveyed” (Wilkins, 1972, p. 111). On the other hand, humans have a colossal ability to deal with indefinite number of words and the phrases they are used, which has never been countable to-date (for a review of psychological processes involved in language acquisition, see Banaruee et al., 2023a). One of the important issues in the realm of L2 research is therefore how vocabulary can be taught and learned effectively (Zimmerman, 1997; Hulstijn, 2001; Folse, 2006; Webb, 2007; Keating, 2008; Schmitt, 2008; Laufer and Rozovski-Roitblat, 2015; Banaruee et al., 2023b). The growing interest in the role vocabulary plays in language acquisition can be testified by the considerable amount of research on vocabulary, each of which addresses a particular issue of vocabulary learning and teaching. E.g., what it means to know a word (Paribakht and Wesche, 1997; Nation, 2001; Schmitt, 2008), which words need to be learnt (Coady and Huckin, 1997; Nation, 2001), how often learners should be exposed to a word (Saragi et al., 1978; Nation, 2001), and which tasks are more effective in vocabulary learning (Hulstijn and Laufer, 2001; Rott et al., 2002). Nonetheless, researchers are still undecided about the mechanisms of language acquisition (Banaruee et al., 2023a) and in particular about the best approaches to learning and teaching vocabulary (Schmitt, 2008).

Various approaches to teaching and learning vocabulary have been proposed in the literature, of which “incidental” and “intentional” vocabulary learning are of great importance (Hulstijn and Laufer, 2001). Intentional vocabulary learning is “the activity aimed at committing lexical information to memory” (p. 11), while incidental learning is a situation in which individuals process new information without intending to store the information in memory. In other words, incidental vocabulary learning refers to “the learning of vocabulary as a by-product of any activity not explicitly geared to lexical learning” (Hulstijn and Laufer, 2001, p. 10). A number of researchers contend that apart from the first few thousand words, vocabulary learning mostly occurs incidentally (Nation and Coady, 1988; Krashen, 1989; Coady, 1997; Paribakht and Wesche, 1997; Huckin and Coady, 1999; Decarrico, 2001; Nation, 2001). This is mainly due to the large number of words and the large amount of time required to teach them (Scott and Nagy, 1997; Banaruee et al., 2023b), and also due to the open-ended unsystematic structure of vocabulary which does not lend itself well to teaching (Mobarge, 1997). However, there are challenges related to time, size, and the way of acquiring vocabulary that is viewed differently according to symbolic and non-symbolic models of language processing and acquisition (see Banaruee et al., 2023a).

One of the fundamental concepts underlying most research on incidental vocabulary learning is the *depth of processing framework* proposed by Craik and Lockhart (1972). It states that “the memory trace can be understood as a byproduct of perceptual analysis and that trace persistence is a positive function of the depth to which the stimulus has been analyzed” (p. 671). Based on this framework, the deeper the processing of a stimulus is, the more elaborate, longer lasting and stronger the traces in memory will be. The hypothesis holds that the retention of information is determined by the depth of its processing, it is processed regardless of how long it remains in the primary memory. In this model, elaboration is the key to learning and retention of vocabulary. One of the main challenges associated with the depth of processing hypothesis is the lack of operationalizable definitions, to employ, grade, classify, and evaluate tasks in terms of their depth of processing and effectiveness. Two frameworks were proposed to operationalize the construct of elaborate processing: Involvement Load Hypothesis (ILH) and Technique Feature Analysis (TFA). Previous research showed the supremacy of TFA to ILH due to its higher explanatory power that leads to the prediction of level of vocabulary acquisition (see Hu and Nassaji, 2016). The aim of the present study was to investigate the predictions made by TFA in relation to the effectiveness of different vocabulary learning tasks.

## 2 Literature review

### 2.1 The involvement load hypothesis

In an attempt to operationalize the construct of elaborate processing, Laufer and Hulstijn (2001) proposed a cognitive-motivational construct of the *task-involvement load* or the *involvement load hypothesis* (ILH), which entails the components of *need*, *search*, and *evaluation*. The presence or absence of each of these components accounts for the involvement load of a task. When none of these components are present in a task, the index 0 is assigned. When they are present in a task, their index could be either 1 or 2 depending on whether they are moderate or strong. A moderate need is when a learner is assigned to look for the meaning of a word, while the strong need is an intrinsic motivation and willingness to look the words up in a dictionary without external imposition from a teacher. A moderate search refers to the situation when a learner recalls the meaning of a word, while its strong mode, involves learners strive to gain more information about the word apart from its meaning, such as part of speech and usage. A moderate evaluation is related to learner’s struggle to compare varying information he has received related to the meanings of a word (e.g., a polysemy, or a homonym), while strong evaluation refers to more pragmatic and contextual-based evaluations the learner has to use a word in the right context (for further explanations see, Laufer and Hulstijn, 2001). The hypothesis suggests that tasks with higher involvement load are more effective for word learning and retention than tasks with lower involvement load. Moreover, no involvement factor takes precedence over another and no particular task type (e.g., input and output) is considered *a priori*, more effective than the other. The degree to which an L2 learner is engaged in cognitive processing does not depend on the type of task, but on the combination of motivational and cognitive dimensions of the task, which they referred to as involvement load (Laufer and Hulstijn, 2001). Another fundamental assertion of the involvement load hypothesis is that time-on-task is an inherent feature of the task that is not amenable to manipulation. However, the potential of the ILH was criticized by Nation and Webb (2011) on the grounds that the framework, which is limited to the dimensions of need, search and evaluation, does not account for other relevant factors that can have an impact on the effectiveness of vocabulary learning activities. Therefore, a different framework, the *Technique Feature Analysis* was proposed.

### 2.2 Technique feature analysis

Nation and Webb (2011) developed the Involvement Load Hypothesis by adding several features that are considered important learning new vocabulary. They proposed a framework that involves a more detailed and elaborate set of criteria and called it the *Technique Feature Analysis* (TFA). It is based on the statement that “the design of the task determines the quality of the learning outcomes” (Nation and Webb, 2011, p. 4). Nation and Webb (2011) added several additional components to the index in order to “meet the dual goals of evaluating and designing techniques.” As a result, the new analysis consists of 18 questions/criteria classified according to psychological conditions that contribute to vocabulary learning. The answer to each question is scored either 0 or 1 with the total score indicating the relative value of an activity, making the highest possible score 18. The TFA includes five components (i.e., motivation, noticing, retrieval, creative use, and retention) and some criteria to assess each component (see Table 1).

**Table 1:** A checklist for technique feature analysis adopted from Nation and Webb (2011, p. 14).

Criteria	Scores	
Motivation		
Is there a clear vocabulary learning goal?	0	1
Does the activity motivate learning?	0	1
Do the learners select the words?	0	1
Noticing		
Does the activity focus attention on the target words? 0	0	1
Does the activity raise awareness of new vocabulary learning?	0	1
Does the activity involve negotiation?	0	1
Retrieval		
Does the activity involve retrieval of the word?	0	1
Is it productive retrieval?	0	1
Is it recall?	0	1
Are there multiple retrievals of each word?	0	1
Is there spacing between retrievals?	0	1
Generation	0	1
Does the activity involve generative use?	0	1
Is it productive?	0	1
Is there a marked change that involves the use of other words?	0	1
Retention		
Does the activity ensure successful linking of form and meaning?	0	1
Does the activity involve instantiation?	0	1
Does the activity involve imaging?	0	1
Does the activity avoid interference?	0	1
Maximum score		18

*Motivation* deals with the clarity of the learning objectives and whether the activity motivates the learner. It is similar to the need component of ILH. The other four components are cognitive components*. Noticing* involves drawing learner’s attention to the unfamiliar words, e.g., by highlighting and sensitizing them so that they realize that there is something to learn. Furthermore, an activity is highly effective if it involves recognition. *Retrieval* refers to the activation of the word form and meaning from the mental lexicon (e.g., through recognition or recall). *Generative use* is related to meeting or using a word in a completely new context. Finally, *retention* refers to the successful establishment of connections between form and meaning (Nation and Webb, 2011). They also analyzed some vocabulary learning activities based on their framework and have created an index for each. Further, they compared the TFA indices of these activities with ILH indices (see Table 2). The two frameworks differ in the different weights they give to individual attention component. The two frameworks may result in differences in predicting which tasks or activities are more effective for teaching and learning vocabulary.

**Table 2:** Comparison of involvement load and technique feature analysis of 10 activities.

Activity	Involvement load	Technique feature analysis
Fill-in-the-blanks	4	8
Find the word in the text	4	8
Write with target words	3	8
True/false	3	6
Reword the sentence	3	6
Multiple-choice on text	3	6
Word cards	3	11
Read and choose definitions	3	6
Reading plus fill in	2	7
Reading with glosses	1	5

Adopted from Nation and Webb (2011, p. 14).

This study aimed to investigate the predictability and explanatory power of the TFA in in relation to the effectiveness of vocabulary learning tasks. Five vocabulary learning tasks were selected that differ in their ranking and the extent to which they promote the different components of the TFA. First, the features of these tasks were examined and their scores were calculated based on the framework. Then, their effectiveness was compared in terms of the participants’ vocabulary knowledge gains. The following two research questions were investigated:

1. Do different tasks with similar levels of TFA index levels lead to similar levels of vocabulary gain?
2. Do different vocabulary tasks with different TFA index levels differ in terms of their contribution to vocabulary gain?

## 3 Method

### 3.1 Participants

Initially, a total of 160 EFL adult learners participated in the study. They were students of English as a Foreign language at Chabahar Maritime University in Iran. The sample included both males and females ranging from 19 to 25 years old (mean age = 21.7, female 61.5%). They were given an Oxford Placement Test and from among them a total of 130 students whose scores were one standard deviation above or below the mean were selected to participate in the study. The mean score for the total population was 71.5. The standard deviation was 11.8. Hence, we excluded the participants whose scores were above 83.3 and those whose scores were below 59.7 from our experimental groups. As a result, the participants in the study were highly homogeneous. Thee participants were randomly selected from six intact classes and the data were collected during their regularly scheduled class periods. In each class, the participants were randomly assigned to one of the five experimental tasks: reading and multiple-choice questions (*n* = 28), reading and choosing definition (*n* = 22), reading and fill-in-the-blanks (*n* = 28), reading and sentence rewording (*n* = 26), and composition writing with the target words (*n* = 26); and those who were excluded from the study received a reading comprehension task as a placebo.

### 3.2 Materials

The researchers had to select tasks consistent with the technique feature analysis framework. Therefore, the tasks proposed in Nation and Webb (2011) were used for comparison: *reading and multiple-choice items* (Task 1, with a TFA score of 6), *reading and choosing definitions* (Task 2, with a TFA score of 6), *reading and fill in the blanks* (Task 3, with a TFA score of 7), *reading and rewording the sentences* (Task 4, with a TFA score of 6) and *composition writing with the target words* (Task 5, with a TFA score of 8). Since all the tasks, with the exception of the task for composition writing, initiated with reading a text, first a reading text had to be developed for the tasks.

#### 3.2.1 The text

The reading passage was adopted from an article in a book on reading comprehension (Richeck, 1993). It was the text previously used by Khoshsima and Eskandari (2017). 551 words comprised the passage dealing with the origins of superstitions. The reading passage was modified to keep the vocabulary within a list of first and second thousand vocabulary apart from the target words (Nation, 1984). The aim of modification was to include the majority of the words in the learners’ experiential vocabulary without simplifying the text. Besides, decreasing the number of unfamiliar words in the text, frees up the cognitive space required to engage with the massage (Joe, 1998). This allows stronger form-meaning connections to be made, so that the target words would be retrievable at a later stage (Craik and Lockhart, 1972; Craik and Tulving, 1975; Nation, 1990; Laufer and Hulstijn, 2001; Banaruee et al., 2023b). Another criterion for modifying the text was the number of occurrences of the individual target words. The passage was revised so that all target words would occur only once.

#### 3.2.2 Target words

Using the Academic Word List (AWL) highlighter (Coxhead, 2000), 20 low frequency words were selected from the text adopted for the study, of which 10 were selected for the research in a pilot study with a similar pool of participants. Moreover, the results of the pretest of the main study showed that the words were completely unknown to the participants. Word length, part of speech, and concreteness/abstractness of the words were not considered in the selection process. Laufer (1997) has amply demonstrated that these three features should not be considered as problematic factors. Words with familiar affixes, or what Laufer (1997) calls “morphologically transparent words” and multi-word lexical items and polysemous words were excluded. This was done for two reasons. Firstly, there was the possibility that some students might know the different meanings of the word and not report all of them. The second reason was related to retention. Learning a different meaning for a word when the subject already knows one or more of its other meanings can help create connections between the new meaning and the known meanings, which ultimately affects retention performance. The target words were six nouns, three were verbs and one adjective. The target words were: *sortie*, *smattering*, *risible*, *guffaw*, *slop*, *betrothal*, *coven*, *berate*, *whiff*, *quandary*.

#### 3.2.3 Tasks

##### 3.2.3.1 Reading and multiple-choice items

Learners performing this task were provided with a text and 10 multiple-choice comprehension questions based on the reading passage. These questions either contained some target words or paraphrased the original sentences in which these target words occurred. Accordingly, successful completion of the questions entailed the understanding of the target lexical items. In the reading passage, the 10 target words whose comprehension was relevant to the task were highlighted in bold print.

##### 3.2.3.2 Reading and choosing definitions

In this task, the target words were highlighted in bold print. After finishing reading the text, the participants had to complete the next task in which they had to choose the correct definition of each target word from four possibilities (similar to Nation and Webb, 2011, p. 322).

##### 3.2.3.3 Reading and fill-in-the-blanks

The participants in this group were given the same text and the same questions as those in *Reading and multiple-choice on text* group. For this group, however, the 10 target words were removed from the text, leaving 10 gaps numbered one to 10. The 10 target words, along with one additional word that did not appear in the original text, were printed in random order as a list on a separate sheet along with their L1 translation, their L2 definition and their grammatical category. The task was to read the text, fill in the 10 gaps with the missing words from the word list, and answer the comprehension questions.

##### 3.2.3.4 Reading and rewording the sentences

In this task, the target words were highlighted in the text. After finishing reading the text, the learners did the task where they were asked to rewrite the sentences with the words in parentheses that were the target words drawn from the text. For example:

*Reword the sentences without changing the meaning. Use an appropriate form of the words in parentheses if necessary.*

*The puzzle was so hard that I was confused. (**quandary**).*

##### 3.2.3.5 Composition writing with the target words

Participants performing this task were required to write a composition about superstitions. This topic was chosen since it was the topic of the text the target words were chosen from. The students were instructed that grammaticality was of a secondary importance and that the clarity of the main idea of the composition as well as the incorporation of the 10 target words would be of the first importance. On a separate page, the target words were provided along with their L1 translation, L2 definition and examples of usage. The part of speech of each word was also specified. Here is an example of the information provided for the word *quandary*:

**Quandary** = (noun)(the Persian translation) giji

*Definition*: state of uncertainty or confusion.

*Example*: He was in a quandary because of the news.

The participants were asked not to copy the example sentences and try to use each target word in a new context. A summary of the tasks along with their TFA indexes are presented in Table 3.

**Table 3:** Four tasks analyzed using technique feature analysis framework.

Criteria	Reading and multiple-choice items	Reading and choosing definitions	Reading and fill-in-the-blanks	Reading and rewording sentences	Composition writing with target words
	Task 1	Task 2	Task3	Task 4	Task 5
Motivation					
Is there a clear vocabulary learning goal?	0	1	1	1	1
Does the activity motivate learning?	1	1	1	0	0
Do the learners select the words?	0	0	0	0	0
Noticing					
Does the activity focus attention on the target words?	1	1	1	1	1
Does the activity raise awareness of new word learning?	0	1	1	1	1
Does the activity involve negotiation?	0	0	0	0	0
Retrieval					
Does the activity involve retrieval of the word?	1	1	0	0	0
Is it productive and retrieval?	0	0	0	0	0
Is it recall?	1	0	0	0	0
Are there multiple retrievals of each word?	0	0	0	0	0
Is there spacing between retrievals?	0	0	0	0	0
Generation					
Does the activity involve generative use?	1	0	1	1	1
Is it productive?	0	0	0	1	1
Is there a marked change that involves the use of other words?	0	0	0	0	1
Retention					
Does the activity successfully link the form and meaning?	0	0	1	1	1
Does the activity involve instantiation?	0	0	0	0	0
Does the activity involve imaging?	0	0	0	0	0
Does the activity avoid interference?	1	1	1	0	1
Total score	6	6	7	6	8

#### 3.2.4 Pretest and posttest

A modified version of vocabulary knowledge scale (Paribakht and Wesche, 1997) developed by Folse (2006) was used for both the pretest and posttest. The test assesses three levels of vocabulary knowledge and can also assess the partial increase in vocabulary knowledge of the test takers. On this modified scale, one point was awarded if the correct meaning was provided (as evidenced by an acceptable English synonym, English definition, L1 translation or definition). One additional point was also awarded if the student could form a correct sentence using the target word. Thus, the participant could receive a score of 0, 1, or 2 for each test item.

### 3.3 The procedures

Two weeks prior to the main study, the participants in the six intact classes took the Oxford Placement Test (OPT). It was conducted during their regular class time. Then those whose score were one standard deviation below the mean or one standard deviation above the mean were selected to participate in the study. Then, we prepared the tasks and revised the main text regarding its length and complexity. The text was reviewed by two native scholars and three lecturers of English language and linguistics at the university. They all confirmed that the words had low frequency.

In addition, the teachers acknowledged that the students would not encounter the words during the semester. All tasks along with the pretest and posttest were administered in the participants’ regular class time on the scheduled review days. Along with the assumption that the participants were not familiar with the target words, all participants were given a vocabulary pretest measuring their knowledge of the target words prior to performing the tasks. Randomization of the experimental tasks in this study occurred within groups. Participants in each class were randomly assigned one of the five experimental tasks. The researchers visited a total of six intact classes and followed the same administration procedure in each class. The students in each class, who did not have the intended level of language proficiency, were given a reading comprehension task as a placebo. Upon the completion of the tasks, the students were unexpectedly given an immediate posttest. The order of the target words in the pretest and posttest was not identical.

## 4 Results

The first research question investigated whether tasks with similar levels of TFA indices lead to a similar increase in vocabulary knowledge. Descriptive statistics for the posttest of the three tasks with the same TFA indices shown in Table 4 reveal that the students who were assigned Task 4 (mean = 3.69) outperformed those who were assigned Task 2 (mean = 3.22) and Task 1 (mean = 2.64). Moreover, the students who were assigned Task 2 had a better performance than those who were assigned Task 1 (see Table 5)

**Table 4:** Descriptive statistics for tasks with similar TFA rankings.

	*N*	Mean	Std. deviation	Std. Error
Sentence rewording (Task 4)	26	3.692	2.412	0.473
Choosing definition (Task 2)	22	3.227	2.384	0.508
Reading and comprehension question (Task 1)	28	2.642	1.603	0.303
Total	76	3.171	2.158	0.247

**Table 5:** One -way ANOVA for tasks with similar TFA rankings.

	Sum of squares	Df	Mean square	F	Sig.
Between groups	14.94	2	7.473	1.632	0.203
Within groups	334.33	73	4.580		
Total	349.27	75			

The ANOVA results for the first research question showed that there was no statistically significant difference between the three tasks with the same TFA indices (*F* = 1.632, sig. = 0.203). The second research question examined whether different vocabulary tasks with varying levels of TFA indices differ in terms of their contribution to vocabulary gain. Descriptive statistics for the five tasks are presented in Table 6.

**Table 6:** Descriptive statistics for tasks with different TFA rankings.

	N	Mean	Std. deviation	Std. Error
Fill-in-the-blank (Task 3)	28	6.142	2.391	0.451
Sentence rewording (Task 4)	26	3.692	2.412	0.473
Choosing definition (Task 2)	22	3.227	2.384	0.508
Reading and comprehension questions (Task 1)	28	2.410	1.610	0.304
Composition writing (Task 5)	26	6.269	2.454	0.481
Total	130	4.380	2.742	0.240

As Table 6 shows, the highest level of vocabulary gain belongs to the group who performed Task 5 (mean = 6.26) followed by Task 3 (mean = 6.14) and the lowest mean score belongs to the group who performed Task 1 (mean = 2.41). A one-way ANOVA was conducted to examine how students differ in their task performance and vocabulary learning gains across the four tasks. The results showed a statistically significant difference (*F* = 16.111, Sig = 0.000), shown in Table 7.

**Table 7:** One -way ANOVA for tasks with different TFA rankings.

	Sum of squares	Df	Mean square	F	Sig.
Between groups	329.92	4	82.48	16.11	0.000
Within groups	639.97	125	5.120		
Total	969.90	129			

A Tuckey *post hoc* was then conducted to examine how individual tasks differed from the others (Table 8). The Tuckey *post hoc* test indicated that the mean score of Task 5 (composition writing) was significantly higher than the mean scores of Task 1, Task 2, and Task 4. The same pattern was also observed for Task 3 (fill-in-the-blank) which was significantly different from Task 1, Task 2, and Task 4. However, Task 3 and Task 5 did not differ significantly from each other.

**Table 8:** Multiple comparison among tasks with different TFA rankings.

(I) Task	(J) Task	Mean difference (I-J)	Std. error	Sig.
Fill-in-the-blank	Sentence rewording	2.450*	0.616	0.001
	Choosing definition	2.915*	0.644	0.000
	Reading and comprehension questions	3.732*	0.604	0.000
	Composition writing	−0.126	0.616	1.000
Sentence rewording	Fill-in-the-blank	−2.450*	0.616	0.001
	Choosing definition	0.465	0.655	0.954
	Reading and comprehension questions	1.281	0.616	0.235
	Composition writing	−2.576*	0.627	0.001
Choosing definition	Fill-in-the-blank	−2.915*	0.644	0.000
	Sentence rewording	−0.465	0.655	0.954
	Reading and comprehension questions	0.816	0.644	0.712
	Composition writing	−3.041*	0.655	0.000
Reading and comprehension questions	Fill-in-the-blank	−3.732*	0.604	0.000
	Sentence rewording	−1.281	0.616	0.235
	Choosing definition	−0.816	0.644	0.712
	Composition writing	−3.858*	0.616	0.000
Composition writing	Fill-in-the-blank	0.126	0.616	1.000
	Sentence rewording	2.576*	0.627	0.001
	Choosing definition	3.041*	0.655	0.000
	Reading and comprehension questions	3.858*	0.616	0.000

^*^The mean difference is significant at the 0.05 level.

Since the results from the analysis of the variance did not provide enough evidence to reject similarity between the effects of varying tasks in enhancing the participants knowledge of vocabulary. A Bayesian test of Welch’s ANOVA was used to assess the subtle differences between the efficacy of individual groups (see Table 9).

**Table 9:** Bayesian estimates of the coefficients^a,b,c^.

Parameter	A-posteriori			95% confidence interval	
	Mode	Mean	Variance	Lower-limit	Upper-limit
Vocabulary task = Reading and multiple-choice items	9,879	9,879	,111	9,225	10,534
Vocabulary task = Reading and choosing definitions	9,523	9,523	,147	8,771	10,274
Vocabulary task = Reading and fill-in-the-blanks	9,750	9,750	,115	9,084	10,416
Vocabulary task = Reading and rewording sentences	10,846	10,846	,124	10,155	11,537
Vocabulary task = Composition writing with target words	10,973	10,973	,110	9,992	11,540

^a^Dependent Variable: posttest. ^b^Model: Type of vocabulary learning task. ^c^Standard reference a priori distributions.

The result of the Bayesian estimates of coefficients and error variance for different types of vocabulary learning tasks showed that the composition writing task yields the highest estimated mean (10,973) and the lowest variance (0.110) among all tasks. This suggests that engaging in composition writing specifically with target words may be particularly effective in enhancing vocabulary acquisition. The second most effective task is reading and rewording sentences (Mean = 10,846). Interestingly, both tasks involve productive language skills. This indicates that tasks aligned with productive skills supersede receptive tasks when practicing vocabulary in the context of foreign language learning. The variance estimates provide insights into the reliability of these estimates. Overall, considering the Bayesian approach and the credible intervals, composition writing appears promising for vocabulary learning.

Table 10 shows the descriptive statistics for the number of correct synonyms or translation that participants provided for each word in the post test. Considering part of speech, no special pattern or difference was found among words in terms of the frequency with which they were recalled. Some nouns such as *coven* and *betrothal* were recalled better than others, and the same trend was observed for the verbs. For instance, the verbs *guffaw* and *slop* were recalled more often than *berate*. Moreover, the verb *guffaw* and the noun *coven* had the same frequency and both were remembered better than other words.

**Table 10:** Percentage of the correct answers given for each word in the posttest.

Words	Sortie	Smattering	Risible	Guffaw	Slop	Betrothal	Coven	Berate	Whiff	Quandary
Part of speech	Noun	Noun	Adj	Verb	Verb	Noun	Noun	Verb	Noun	Noun
Percentage	37	41.3	54.34	63.04	60.86	52.17	63.04	30.43	34.78	23.91

## 5 Discussion

The experiment examined whether or not tasks with similar and different TFA indices led to similar or different levels of target vocabulary knowledge gain in EFL learners with the same level of proficiency. The results related to the first research question supported the TFA claim that tasks with similar ranking indices lead to similar levels of vocabulary knowledge growth. Furthermore, the results of the second question were partially in accord with the predictions made by TFA that tasks with higher TFA indices would lead to better vocabulary retention. Even though no significant difference was observed between different tasks of learning vocabulary based on the results of the analysis of variance, the subtle differences in the mean values indicated that tasks related to the productive language skills, composition writing and rewording sentences, were more effective in enhancing the participants knowledge of the novel words. Considering Nation and Webb’s (2011) framework, fill-in-the-blank and writing tasks differ in some aspects such as *motivation* and *generative use*. According to Nation and Webb (2011), activities that present a challenge to the learner are to motivate learning, which in turn will lead to better vocabulary acquisition, which can be partially confirmed based on our results. Thus, tasks such as Fill-in-the-blank, multiple-choice, and selecting definitions differ from tasks which involve sentence or language production. Nonetheless, a composition writing task is no guarantee of a motivation boost, rather positively generative. On the other hand, composition writing involves using the target words in a completely new and coherent self-constructed context, which is highly demanding and involves deeper processing of the word (Nation and Webb, 2011), a process that Zou (2016) considered as a strong evaluation of a word and also as the most effective factor in vocabulary acquisition in his analysis of the components of the *Involvement Load Hypothesis*. Fill-in-the-blank task, as Nation and Webb (2011) show, involves this generative use but not a productive one, and they consider it a less demanding task. Further, it is considered a *moderate evaluation* since it involves comparison among different words but not using them in a new context (see Laufer and Hulstijn, 2001; Zou, 2016).

*Exposure* is one of the factors that have a positive effect on vocabulary acquisition (Banaruee et al., 2023a), which is present in both fill-in-the-blank and composition writing tasks (Nation, 2001; Folse, 2006; Peters, 2012). Folse (2006) argues that repetition is more important than depth of processing. Repeated exposure to novel words can consolidate the form-meaning connection of words (Hulstijn, 2001; Nation, 2001; Laufer, 2005). This is highly argued in studies related to meaning formation and metaphor where phenomena such as conventionalization of meaning (e.g., Bowdle and Gentner, 1999; Khatin-Zadeh et al., 2019), structural-mapping (e.g., Gentner, 1983; Khatin-Zadeh et al., 2022), and class-inclusion (e.g., Glucksberg and Keysar, 1990; Khatin-Zadeh et al., 2017) are investigated. These models discuss not only how words but the way concepts are processed in our language acquisition and processing. During fill-in-the-blank activity, the participants have to compare the words with each other and it continues until all the blanks are filled, a process which Folse (2006) considered as a deep processing of the words. This also involves a structural-mapping and class-inclusion process that is used to find a salient semantic feature which can be mapped between different domains used in a statement (Khatin-Zadeh et al., 2017, 2019). As a result, the learner is confronted to each target word several times. In the case of composition writing task, the additional exposure to target words happens during *pre-task planning* (Zou, 2016). The participants had to use the target words in the original context, and before writing anything down on the paper, “they created potential scenarios incorporating the target words in a virtual mental space” (Zou, 2016, p. 68). Hence, the participants practiced the target words twice: virtually during pre-task planning and in practice when writing the words down on the paper. Despite the fact that there were similarities between the features of some of the tasks regarding having glossed target words, this aspect of vocabulary learning was not a predictor of vocabulary gain (contrary to previous research, see Schmitt, 2008). Glossing leads to successful acquisition of form-meaning link that is the primary and most essential lexical aspect to be acquired (Schmitt, 2008). L2 definitions or synonyms provide learners with additional exposure to the target language (Joyce, 2018). Furthermore, the deployment of L1 in L2 learning can provide a shortcut to vocabulary acquisition (Scott and De la Fuente, 2008) since languages have conceptually a lot in common (Swan, 1997; Khatin-Zadeh et al., 2023; Banaruee et al., 2024). Many studies argue that glosses help learners learn new lexical items effectively (see, e.g., Bowles, 2004; Cheng and Good, 2009). Learner’s attention to the glossed words promotes learning (Nation, 2001). Moreover, it helps students deal with insufficient contextual clues when learning new words. Considering the discussed similarities and differences which exist between fill-in-the-blank and composition writing tasks in terms of motivation, generative use, evaluation, kind of exposure, and glossing, it is presumable that both tasks have their own merits in one way or another. Previous research disagrees on which of these activities is more effective in supporting vocabulary learning (Laufer, 2003; Folse, 2006; Keating, 2008). In contrary to Laufer’s (2003) results that showed that reading plus a written task led to better retention of meaning compared to reading plus a blank-filling task, our result did not show this difference. Nevertheless, results of the delayed posttest in Laufer (2003) showed a decrease in the benefits of the written task. Such contradictory results urge the need for further research to compare different tasks according to TFA framework and the effectiveness of fill-in-the-blank and composition writing tasks in vocabulary acquisition. Our results showed that fill-in-the-blank and composition writing tasks promote the learning of vocabulary among adult EFL learners to a similar degree. However, regardless of the potential effectiveness of each of these two tasks, a teacher may choose any of them according to the syllabus, learning goals, time and most importantly students’ needs, their learning styles and individual preferences. Learning and cognitive styles have shown to play a crucial role in success in reading tasks (Askari et al., 2017; Banaruee et al., 2022), and academic achievement (Yarahmadzehi and Banaruee, 2017) among EFL learners.

## 6 Conclusion

The study investigated and compared the effect of task-type on vocabulary acquisition. Overall, the results of the study provided partial support for the claims made by the *Technique Feature Analysis* framework. Yet, the findings suggested that productive tasks; composition writing and rewording sentences lead to a higher gain of vocabulary knowledge among adult EFL learners. The findings yielded by the study can provide both teachers and material developers with insights as to the effectiveness of the kinds of tasks they use or develop in vocabulary acquisition. The TFA framework can be a good foothold to help them design tasks conducive to better and more vocabulary knowledge gain. Moreover, this study could possibly lay the ground for a great deal of research to touch on the effect of different vocabulary-learning tasks on the amount and various aspects of vocabulary acquisition; i.e. investigating both the quantity and quality of vocabulary learning. There are few points that should be considered when interpreting the results of this study. The present study provided the participants with only one exposure to the target words and only examined the short-term retention of the target words. Moreover, the researchers failed to control time on task which may intervene and affect the yielded results. Longer time on task or longer exposure to the target words might affect the results. The participants in this study were of intermediate level of proficiency. The study might produce different results with participants of other levels of proficiency. Finally, the kind of test used in this study demanded that the participants recall the meaning of the target words. Research with different kind of tests especially those which involve both recognition and recall might yield different findings.

## Data availability statement

The raw data supporting the conclusions of this article will be made available by the authors, without undue reservation.

## Ethics statement

The studies involving humans were approved by Ethic Committee Name: Bartar Language Academy. Approval code: BLA14012. The studies were conducted in accordance with the local legislation and institutional requirements. The participants provided their written informed consent to participate in this study.

## Author contributions

ZE: Conceptualization, Data curation, Methodology, Supervision, Writing – original draft, Writing – review & editing. OK-Z: Data curation, Formal analysis, Investigation, Methodology, Writing – original draft, Writing – review & editing. DF: Conceptualization, Formal analysis, Funding acquisition, Visualization, Writing – review & editing. HB: Data curation, Investigation, Methodology, Resources, Writing – review & editing.
